# Supplemental Oxygen during Exercise Training in Chronic Obstructive Pulmonary Disease

**DOI:** 10.1249/MSS.0000000000003782

**Published:** 2025-06-12

**Authors:** SARA FAGGIAN, FRANCESCA BATTISTA, MARCO VECCHIATO, RICHARD CASABURI, MARGARETA EMTNER, NICOLA BORASIO, MICHAEL STUDNICKA, ANDREA ERMOLAO, JOSEF NIEBAUER, DANIEL NEUNHAEUSERER

**Affiliations:** 1 Sports and Exercise Medicine Division, Department of Medicine, University of Padova, Padova, ITALY; 2 Respiratory Research Institute, The Lundquist Institute for Biomedical Innovation at Harbor-UCLA Medical Center, Torrance, CA; 3 Department of Medical Sciences, Respiratory-, Allergy- and Sleep Research, Uppsala University, Akademiska sjukhuset, Uppsala, SWEDEN; 4 University Clinic of Pneumology, Paracelsus Medical University of Salzburg, Salzburg, AUSTRIA; 5 University Institute of Sports Medicine, Prevention and Rehabilitation and Research Institute of Molecular Sports and Rehabilitation Medicine, Paracelsus Medical University of Salzburg, Salzburg, AUSTRIA

**Keywords:** CHRONIC OBSTRUCTIVE PULMONARY DISEASE, PULMONARY REHABILITATION, EXERCISE CAPACITY, CARDIORESPIRATORY FITNESS, VENTILATORY LIMITATION

## Abstract

**Introduction:**

Chronic obstructive pulmonary disease is a leading cause of mortality worldwide and a debilitating condition that leads to years of poor quality of life. Physical exercise training is an evidence-based treatment well documented to improve these outcomes as well as morbidity, dyspnea, and functional capacity. Moreover, scientific evidence from pooled analyses currently provides equivocal evidence for oxygen supplementation to overcome ventilatory limitations during exercise training, with several studies reporting no additional benefits when compared with training in room air. However, when individually analyzing the underlying studies from an exercise physiology perspective, some critical aspects arise.

**Purpose:**

This review aims to systematically investigate and highlight the impact of patients’ characteristics, exercise-induced desaturation, oxygen delivery, influence of breathing conditions during exercise testing and prescription, outcome-training specificity, exercise intensity and modality, and progressive work rate adjustments over the course of the training intervention.

**Methods:**

The research methodology is based on a literature search of the available evidence starting from the published systematic reviews and meta-analyses, and integrating available original articles from the respective reference lists.

**Results:**

Although evidence is still limited, supplemental oxygen might be specifically useful for certain responding patients and in specific clinical conditions, when high-intensity training is performed, thereby increasing exercise tolerance in order to improve training adaptations and thus peak exercise capacity/endurance.

**Conclusions:**

Future well-designed clinical trials may better implement these methodological training principles in their study design and investigate if advantages from normoxic and hyperoxic exercise training can be weighed, showing how, when, and in which patients supplemental oxygen could be best used in order to reach predefined training goals in pulmonary rehabilitation.

Chronic obstructive pulmonary disease (COPD) is one of the leading causes of morbidity and mortality worldwide, but also a debilitating disease that leads to years, if not decades, of poor quality of life due to the hallmark symptom of shortness of breath (1). Although symptoms are often poorly controlled by medical therapy, an evidence-based treatment option remains underused: physical exercise training. Indeed, several studies have shown that rehabilitative exercise improves symptoms, exercise capacity, cardiorespiratory fitness, and quality of life as well as reduces mortality risk (2). Studies comparing exercise training strategies and training adjuncts such as supplemental oxygen (O_2_), however, remain scarce (3,4) but are needed to allow individually tailored exercise prescriptions to optimize treatment effects.

## Ventilatory limitations affect exercise tolerance and training adaptations

In COPD, the impact of training interventions is often limited by patients’ reduced exercise tolerance, resulting in low training intensities, particularly due to ventilatory limitations to exercise. Therefore, several studies aimed at increasing the training effect by providing supplemental O_2_ during endurance exercise training in patients with and without exertional hypoxemia with conflicting results (3,4).

Supplemental O_2_ lends itself to be applied in patients with COPD during exercise due to its acute positive effects on maximal exercise performance secondary to increased arterial partial O_2_ pressure, reduced pulmonary artery pressure, and particularly through carotid body inhibition leading to a more efficient breathing pattern, less dynamic hyperinflation, work of breathing, and thus dyspnea. O_2_ supply to peripheral muscles tends to increase, which improves peripheral O_2_ uptake and lowers blood lactate accumulation, probably affecting peripheral muscle fatigability (5–9). Supplemental O_2_ should enable patients to exercise at higher intensity and for longer duration. However, evidence garnered from published training intervention studies and also recent guidelines provides weak or absent support for O_2_ supplementation during exercise training in COPD (3,4,8,10,11). Indeed, in pooled analyses, O_2_ supplementation seems to provide no additional benefit compared with training while respiring room air. However, this lack of support deserves examination, as some critical aspects arise when analyzing individual studies from an exercise physiology perspective. In particular, it is important to consider population characteristics, O_2_ delivery, the monitored outcomes, exercise work rate and modality, strategies used to prescribe exercise intensity, and adjustment of exercise work rate during the training intervention. This review aims to display relevant features of previous studies that may influence the ability of supplemental O_2_ to improve functional capacity in an exercise training program. Furthermore, the objective is to find training strategies that could improve the utility of supplemental O_2_ in rehabilitative exercise programs. Therefore, we propose the novel hypothesis that the most effective utilization of supplemental O_2_ in patients depends on specific training goals and patient’s objectives.

The research methodology is based on a literature search of the available evidence starting from the published systematic reviews and meta-analyses (see Table 1), and integrating available original articles from the respective reference lists. Moreover, each selected original article has been analyzed with a standardized approach categorizing information regarding participant characteristics (see Table 2), impact of training interventions with or without supplemental O_2_ on several outcome measures (see Table 3), and cornerstone characteristics related to exercise prescription with and without supplemental O_2_ (see Table 4).

**TABLE 1:** Pooled analyses of current evidence.

Reference	Conclusions
Freitag et al., 2020 (4)	“No superior long-term adaptations on physical fitness, functional capacity or patient-reported well-being were found, questioning the role of this method as a personalized medicine approach.”
Hasegawa et al., 2023 (12)	“The available data do not support the use of supplemental oxygen for dyspnea relief in patients with advanced progressive illness, except for dyspnoea during exercise.”
Nonoyama et al., 2007 (3)	“This review provides little support for oxygen supplementation during exercise training for individuals with COPD, but the evidence is very limited.”
Puhan et al., 2004 (13)	“Data from five trials showed that supplemental oxygen during exercise did not have clinically meaningful effects on health-related quality of life, while improvements of exercise capacity may be even larger for patients exercising on room air.”

Conclusions of systematic reviews and meta-analyses regarding exercise training interventions with supplemental oxygen in patients with COPD.

**TABLE 2:** Participant characteristics in studies included in the systematic reviews.

References	Sample Size, *n* (F/M)	Subjects with EID (%)	Age (yr)		COPD Grade	FEV_1_ (% Pred)	
			Air Group	O_2_ Group		Air Group	O_2_ Group
Alison et al., 2018 (14)	95 (53/44)	100	69 ± 8	69 ± 7	Moderate to severe	45 ± 16	47 ± 17
Dyer et al., 2012 (15)	47 (30/17)	100 (100% oxygen responders)	70 ± 7	68 ± 8	Severe	44 ± 11	39 ± 16
Emtner et al., 2003 (9)	30 (11/19)	0	67 ± 10	66 ± 7	Severe	38 ± 8	35 ± 10
Fichter et al., 1999 (16)	10 (10/0)	na	59 ± 7	58 ± 11	Moderate to severe	46 ± 27	41 ± 8
Garrod et al., 2000 (17)	26 (7/19)	100	52–81	54–77	Severe	35 ± 10	29 ± 10
Neunhäuserer et al., 2016 (18)	29 (8/21)	50	64 ± 6 (crossover trial)		Severe	46 ± 9 (crossover trial)	
Ringbaek et al., 2013 (19)	45 (24/21)	100	69 ± 8	69 ± 10	Severe	31 ± 12	33 ± 16
Rooyackers et al., 1997 (20)	24 (na)	100	59 ± 13	63 ± 5	Severe	38 ± 11	29 ± 7
Scorsone et al., 2010 (21)	20 (6/14)	na	68 ± 7	67 ± 9	Moderate to severe	50 ± 12	47 ± 10
Spielmanns et al., 2015 (22)	36 (na)	0	64 ± 8	65 ± 9	Moderate to severe	43 ± 12	44 ± 10
Wadell et al., 2001 (23)	20 (10/10)	na	69 (60–72)	65 (52–73)	Moderate to severe	52 (24–66)	39 (23–59)

na, not available or not clearly reported.

**TABLE 3:** Impact of training interventions with or without oxygen supplementation on exercise tolerance and quality of life–related measures in patients with COPD.

References	Outcome		Improved Primary and Secondary Outcomes		
			Within-Group Difference from Baseline		Between-Group Difference
			Air Group	O_2_ Group	O_2_ vs Air Group
Alison et al., 2018 (14)	ESWT	Time	+	+	=
	CRQ	Total	+	+	=
Dyer et al., 2012 (15)	ESWT	Distance	+	+	↑
	HADS	Anxiety	−	−	=
		Depression	−	−	=
	CRQ	Mastery subscale	−	−	↑
		Dyspnea subscale	−	−	=
		Fatigue subscale	−	−	=
Emtner et al., 2003 (9)	Constant work rate test (75% peak work rate—air)	Time	+	+	↑
	Maximal CPET (cycle ergometer)	Absolute peak work rate	+	+	=
		Absolute V̇O_2peak_	−	−	na
	CPET (cycle ergometer) isotime	HR	−	+	↑
		Absolute V̇O_2_	−	+	na
		Tidal volume	−	+	↑
		Respiratory rate	−	+	↑
	CRQ	Total	+	+	=
		Dyspnea	+	+	=
		Emotional functioning	+	+	=
		Fatigue	+	+	=
		Mastery subscale	+	+	↑
	SF-36	General health	−	+	↑
		Vitality	+	+	=
		Physical functioning	−	+	=
		Role physical	−	+	=
Fichter et al., 1999 (16)	Maximal CPET (cycle ergometer)	Ventilation	−	−	na
		Respiratory rate	−	−	na
		Tidal volume	−	−	na
		V̇O_2peak_	−	−	na
		Lactate	−	−	na
		HR_max_	−	−	na
		Peak work rate	+	−	na
	CPET (cycle ergometer) isotime	Ventilation	−	−	na
		Respiratory rate	−	−	na
		Tidal volume	−	−	na
		Absolute V̇O_2peak_	−	−	na
		Lactate	+	−	na
		HR	−	−	na
Garrod et al., 2000 (17)	ISWT—air	Distance	−	−	=
		Borg dyspnea score	−	+	↑
	CRQ	Total	−	−	=
	HAD		−	−	=
	LCADL		−	−	=
Neunhäuserer et al., 2016 (18)	Maximal CPET (cycle ergometer)	Absolute peak work rate	+	+	↑
		PPO relative to body mass	+	+	↑
		V̇O_2peak_ relative to body mass	+	+	=
		Expected relative exercise capacity	+	+	↑
		HR_max_	+	+	=
		Lactate	+	+	=
	HADS	Anxiety	+	+	=
		Depression	+	+	=
Ringbaek et al., 2013 (19)	ESWT	Time	+	+	=
		Distance	+	+	=
	SGRQ		−	−	
Rooyackers et al., 1997 (20)	Maximal CPET (cycle ergometer)—air	Absolute peak work rate	+	−	=
	Constant test (cycle ergometer) at 65% peak work rate	Time	−	−	=
	6MWT	Distance	+	+	=
	CRQ	Total	+	+	=
		Mastery subscale	+	+	=
Scorsone et al., 2010 (21)	Maximal CPET (cycle ergometer)	Absolute peak work rate	+	+	=
	Constant-load test (80% peak work rate)	Time	+	+	=
	Constant-load test (isotime 100% pretraining)	HR	+	+	=
		Dyspnea	+	+	=
Spielmanns et al., 2015 (22)	Maximal CPET (cycle ergometer)—air	Absolute peak work rate	+	+	=
		V̇O_2peak_ relative to body mass	−	+	na
		Ventilation	+	+	na
	6MWT	Distance	+	+	na
	SF-36	General health	+	+	na
		Vitality	+	+	na
		Physical functioning	+	+	na
		Mental health	+	+	na
		Role physical	−	−	na
		Role emotional	−	−	na
		Bodily pain	−	−	na
		Social functioning	+	+	na
Wadell et al., 2001 (23)	6MWT on a nonmotorized treadmill—air	Distance	+	+	=

+: the given group improved statistically significantly from baseline; −: the given group did not improve statistically significantly from baseline; ↑: a statistically significant difference between the O_2_ and air groups was found; =: no statistically significant differences between the O_2_ and air groups were found.

CPET, cardiopulmonary exercise test; CRQ, Chronic Respiratory Disease Questionnaire; ESWT, Endurance Shuttle Walk Test; HADS, Hospital Anxiety and Depression Scale; HRQoL, health-related quality of life; ISWT, Incremental Shuttle Walk Test; LCADL, London Chest Activity of Daily Living Scale; na, not available, not clearly reported or not applicable; SGRQ, St. George’s Respiratory Questionnaire; SIFT, Surrey Information on Function Tool.

**TABLE 4:** Cornerstone characteristics related to exercise prescription with and without supplemental oxygen in patients with COPD.

References	Exercise Training						
	Type	Work Rate				(Time × Frequency) × Weeks	Oxygen Flow Delivery (L·min^−1^)/FiO_2_ (%)
		Target Intensity	Exercise Prescription	Air Group	O_2_ Group		
Alison et al., 2018 (14)	Endurance (**treadmill** + *bike*)	Moderate continuous endurance training (**20′** + *20′*)	Parameter: **80% average 6MWT speed**, *60% estimated peak work rate* Gas-specific: No (air) Outcome-specific: **Yes**, *No* Progression: Yes; “work rate was increased according to symptoms so that dyspnea or rate of perceived exertion was at “moderate” to “somewhat severe” level”	**↑ D**yspnea**: 3.7 ± 1.3** **↑ RPE Fatigue: 3.9 ± 1.2** **METs: 62.1 ± 9.4***^a^* *D*yspnea*: 3.5 ± 0.9* ***↑** RPE Fatigue: 4.0 ± 1.1* **↑** *METs: 60.3 ± 12.7^a^*	**D**yspnea**: 3.2 ± 1.1** **RPE Fatigue: 3.1 ± 1.1** **METs: 64.3 ± 9.5***^a^* *D*yspnea*: 3.4 ± 1.1* *RPE Fatigue: 3.5 ± 1.1* *METs: 63.8 ± 13.9^a^*	(30–40′ × 3) × 8	5
Dyer et al., 2012 (15)	Endurance (**walk** + *bike*) + resistance	**High continuous training**	Parameter: 85% predicted V̇O_2peak_ during ISWT and intensity as high as possible to maintain SpO_2_ above baseline nadir SpO_2_ during ESWT. Gas-specific: No (air) Outcome-specific: **Yes**–*No* (**ESWT**) Progression: na	na	na	(5) × 6–7	2, 4, 6
Emtner et al., 2003 (9)	Endurance (*bike*)	*High continuous endurance training (35′)*	Parameter: *75% peak work rate CPET* Gas-specific: No (air) Outcome-specific: *Yes, bike submaximal intensity* Progression: *Yes; “subsequently adjusted considering the subject’s d*yspnea *and fatigue sensations”*	**↑** *Work rate: 52 ± 22 W (96% peak work rate)* **↑** *D*yspnea*: 4.2 ± 0.5* *Leg fatigue: 4.4 ± 0.6* *Lactate: 4.0 ± 0.1 mq·L^−1^* *HR: 120.9 ± 2.2^a^*	*Work rate: 62 ± 19 W (138% peak work rate)* *D*yspnea*: 5.1 ± 0.5* *Leg fatigue: 4.8 ± 0.4* *Lactate: 4.6 ± 0.2 mq·L^−1^* *HR: 126.6 ± 2.3^a^*	(45′ × 3) × 7	3
Fichter et al., 1999 (16)	Endurance (*bike*)	*High continuous endurance training*	Parameter: *80% peak work rate CPET* Gas-specific: No (air) Outcome-specific: *Yes* Progression: *No*	na	na	(45′ × 5) × 4	35%
Garrod et al., 2000 (17)	Endurance (**walk** + *bike*) + resistance	**High (?) training** ***“They exercised for as long as they could”***	Parameter: **80% V̇O**_**2peak**_ **during ISWT air**, *unloaded cycling* Gas-specific: No (air) Outcome-specific: **Yes**–*No* (ISWT) Progression: Yes–No, “the Borg breathlessness score was used to monitor intensity”	na	na	(60′ × 3) × 6	4
Neunhäuserer et al., 2016 (18)	Endurance (*bike*) + Resistance	*High interval endurance training (1′ high: 2′ low × 7)* High resistance training (1 × 8–15 reps to failure)	Parameter: *70%–80% peak work rate CPET* Gas-specific: Yes Outcome-specific: *Yes, bike maximal intensity* *Progression: Yes; “workload was progressively increased whenever a patient’s HR decreased”*	na *(Exercise prescription adapted to room air testing)*	na *(Exercise prescription adapted to testing with supplemental O_2_)*	(30′ × 3) × 6	10
Ringbaek et al., 2013 (19)	Endurance (**walk** + *bike*)	**High continuous endurance training**	Parameter: 85% predicted V̇O_2peak_ during ISWT. Gas-specific: No (air) Outcome-specific: **Yes**–*No* (**ESWT**) Progression: No	na	na	(30′ × 5) × 7	2
Rooyackers et al., 1997 (20)	Endurance (*bike + rowing*) + resistance training + ADLs	*Low-moderate interval training (2′ exercise:2′ rest × 5)*	Parameter: *30%–40% peak work rate, SpO_2_ >90%* Gas-specific: No (air) Outcome-specific: *No (maximal bike CPET, submaximal moderate intensity, 6MWT)* Progression: *Yes, “after the first week, the exercise intensity was gradually increased as tolerated by the patients”*	*Last week of interval cycle exercise training work rate = 114 W (32% peak work rate—air)*	*Last week of interval cycle exercise training work rate = 124 W (43% peak work rate—air)*	((25′ + 55′) × 5) × 10	4
Scorsone et al., 2010 (21)	Endurance (*bike*)	*High continuous endurance training*	Parameter: *80% peak work rate CPET, d*yspnea*, or leg Borg score 5/10, oxygen saturation* Gas-specific: No (air) Outcome-specific: *Yes (maximal bike CPET, submaximal moderate intensity, 6MWT)* Progression: *Yes–No, “If the target time was achieved with dyspnea and leg Borg score* ≤*5/10, the load was increased by 10 W at the next session. The final target workload of 80% of maximum work was gradually achieved within 2 to 3 weeks”*	*Work rate × time: 1001 ± 174^a^* *Sessions to reach 63% maximum training workload:* *7.2 ± 0.3*	*Work rate × time: 874 ± 181^a^* *Sessions to reach 63% maximum training workload:* *7.1 ± 0.2*	(40′ × 3) × 8	40%
Spielmanns et al., 2015 (22)	Endurance (*bike*)	*Moderate continuous training (30′)* *High interval (1′ very high:4′ moderate ×6) training*	Parameter: *60%–125% peak work rate CPET* Gas-specific: Yes Outcome-specific: *Yes*–**No** (*bike maximal intensity*, **6MWT**) Progression: *Yes, “Training intensity was assessed according to the baseline and 12-week training cycle tests and progressively increased every 3 weeks”*	na	na	(30′ × 3) × 24	4
Wadell et al., 2001 (23)	Endurance (**treadmill**)	**High interval training (2–3′ high speed:2–3′ low speed × 5)**	Parameter: **overall mean RPE 17/20 and dyspnea 7/10, oxygen saturation > 90%.** Gas-specific: No (air) Outcome-specific: **Yes, treadmill 6MWT** Progression: **Yes, “the intensity of the sessions with variation of treadmill speed and inclination was individualized with respect to the patients’ saturation and their subjective ratings of dyspnea and perceived exertion”**	**From first to last week, total distance walked increased by 1952 (50%)**	**From first to last week, total distance walked increased by 2173 (43%)**	(30′ × 3) × 8	5

Treadmill and walk-related exercise is denoted by bold text, and cycle ergometer-related exercise is denoted by italic text. Gas-specific refers to prescribing an adequate exercise training intensity by administering the relevant respired gas during the pretraining assessment; ↑: a statistically significant difference between the O_2_ and air group was found.

*^a^* Data from figures were extracted using an online tool (WebPlotDigitizer; https://automeris.io/WebPlotDigitizer/).

CPET, cardiopulmonary exercise test; ESWT, Endurance Shuttle Walk Test; ISWT, Incremental Shuttle Walk Test; na, not available or not clearly reported; RPE, rating of perceived exertion; 6MWT, 6-min walking test.

Table 1 shows the conclusions of systematic reviews and meta-analyses regarding exercise training interventions in which supplemental O_2_ was administered to patients with COPD (3,4,12,13).

Overall, such pooled (meta-)analyses currently provide little to no support for O_2_ supplementation during exercise training because of the lack of demonstrated positive effects in some studies; this has influenced recent guidelines (10,11). But do these pooled analyses correctly assay the studies’ components they reviewed? Indeed, although scientific knowledge is increasing, current evidence specifically investigating methodological principles of applying supplemental O_2_ during exercise training is limited. Therefore, this brief review aims to analyze the original evidence behind those pooled (meta-)analyses, providing novel insights that may affect related outcomes and thus future best practice.

Participant characteristics are included in Table 2.

Table 3 displays the (i) impact of exercise training on functional capacity and quality of life according to the training intervention with or without supplemental O_2_ and (ii) difference between supplemental O_2_ and room air breathing groups (9,14–23).

Furthermore, it is important to consider the exercise prescriptions and related training interventions incorporated into these studies (9,14–23) (Table 4).

## Exercise-induced oxygen desaturation

Randomized controlled trials (RCTs) on this topic mainly included subjects with exercise-induced desaturation (EID). Only Spielmanns et al., (22) and Emtner et al. (9) excluded those patients, whereas Dyer et al. (15) recruited only subjects with EID also showing an improvement in Endurance Shuttle Walk Test of >10% with supplemental O_2_ (24). Indeed, EID seems to play a key role in deciding whether supplemental O_2_ is considered during training. Of relevance, Neunhäuserer et al. (18) observed greater O_2_ effects on peak work rate in patients with EID compared with those nondesaturating. Moreover, EID and supplemental O_2_ were identified as opposing determinants of training-induced muscle gain (18). Indeed, the known heterogenous functional and structural muscle adaptations during training interventions in COPD might be also determined by EID (25).

Another O_2_-related determinant is the subject’s improvement in exercise tolerance in response to O_2_ supplementation during exercise (24). When only “responders” with EID were included, Dyer et al. (15) found greater walking distance improvements in the O_2_ group versus the air group. Indeed, in the study by Ringbaek et al. (19), despite not finding a statistically significant difference in exercise tolerance improvement between the two groups, a clinically relevant increase in walking time after training was only revealed in “responders,” as defined at baseline. However, in most studies, the impact of training and supplemental O_2_ in acute “responders” versus “nonresponders” was not analyzed.

## Oxygen delivery

When examining available clinical trials, it is notable that O_2_ delivery was generally low in concentration and flow rate (2–5 L·min^−1^ or 35%–40% FiO_2_) (9,14–17,19–23), as also reported by Nonoyama et al. (3), even though a dose–response relation has been described in dynamic hyperinflation reduction (26). Indeed, it has been hypothesized that providing a higher O_2_ concentration may have improved training outcomes (3). An exception was the study of Neunhäuserer et al. (18), where exercise training was performed administering 10 L·min^−1^ of O_2_ via nasal cannula; in this double-blind, crossover study, no O_2_-related adverse events were reported, and greater peak exercise capacity improvement was observed in the supplemental O_2_ group when performing high-intensity interval training.

## The gas respired affects exercise prescription

This consideration refers to prescribing an adequate exercise training intensity by administering the relevant respired gas during the pretraining assessment. If training is performed with supplemental O_2_, exercise testing for exercise prescription should be performed with supplemental O_2_ to ensure optimal training intensities. Performing this assessment while breathing air may lead to “under-dosed” training intensity when supplemental O_2_ is administered during the training program. Supporting this concept, it has been shown that the acute effect of O_2_ during maximal testing leads to improved peak work rate, whereas peak heart rate (HR) and ventilation are not influenced (9,15,17,19,20,27–32).

Accordingly, considering this added value of O_2_ on exercise capacity, it seems that exercise prescription should be adjusted to the enhanced exercise tolerance. Practical examples are reported in Tables 5a and 5b.

**TABLE 5A:** Practical example of exercise intensity prescription according to the pretraining assessment with or without supplemental oxygen.

Reference	Peak Work Rate at Exhaustion (W)	Exercise Prescription at 70% Peak Work Rate (W)
Helgerud et al., 2009 (28)	27	19
	39 (∆O_2_–Air: +44%)↑	27
Exercise prescription at 19 W would correspond to 70% of peak work rate for subjects without oxygen but 49% of peak work rate for subjects training with O_2_, leading to “underdosed” exercise intensity in the O_2_ group		
Neunhäuserer et al., 2017 (27)	100	70
	107 (∆ O_2_–Air: +7%)↑	75
Exercise prescription at 70 W would correspond to 70% of peak work rate for subjects without oxygen but 65% of peak work rate for subjects training with O_2_, leading to “underdosed” exercise intensity in the O_2_ group		
Rooyackers et al., 1997 (20)	70	49
	82 (∆ O_2_–Air: +17%)↑	57
Exercise prescription at 49 W would correspond to 70% of peak work rate for subjects without oxygen but 60% of peak work rate for subjects training with O_2_, leading to “underdosed” exercise intensity in the O_2_ group		

↑: a statistically significant difference between O_2_ and air test was found.

**TABLE 5B:** Practical example of exercise volume prescription according to the pretraining assessment with or without supplemental oxygen.

Reference	Time to Exhaustion (min)
Emtner et al., 2003 (9)	Air: 6.6
	Oxygen: 11.8 (∆ O_2_–Air: +79%)↑
O’Donnell et al., 1997 (30)	Air: na
	Oxygen: na (∆ O_2_–Air: +35%)↑
Rooyackers et al., 1997(a) (20)	Air: 6.5
	Oxygen: 11.4 (∆ O_2_–Air: +75%)↑
Rooyackers et al., 1997(b) (20)	Air: 4.5
	Oxygen: 11.0 (∆ O_2_–Air: +144%)↑

↑: a statistically significant difference between O_2_ and air test was found. Rooyackers et al., 1997(a): patients randomly allocated to general exercise training while breathing room air; Rooyackers et al., 1997(b): patients randomly allocated to general exercise training while breathing supplemental oxygen.

na, not available or not clearly reported.

Specific adaptation of exercise prescription based on the respired gas was performed only by Neunhäuserer et al. (18) and presumably Spielmanns et al. (22). Neunhäuserer et al. found a statistically significant O_2_ effect on peak exercise capacity, whereas Spielmanns and colleagues reported increased peak work rate and peak oxygen uptake (V̇O_2peak_) improvements in both the air and O_2_ groups without assessing between-group differences. Furthermore, other breathing conditions must also be considered for exercise prescription, such as the impact of a face mask or mouthpiece as used during cardiopulmonary exercise testing. Its demonstrated influence on exercise tolerance might lead to under-dosing of exercise intensity during training in COPD (27).

## Matching training strategy with outcome measures

This concept refers to training based on predefined objectives, goals, or outcomes. For example, if the main outcome is peak work rate or V̇O_2peak_, as assessed during an incremental exercise test, then high-intensity training is the preferred modality. Of the evaluated RCTs, some did not conduct their exercise intervention based on this principle (20). Moreover, peak work rate has been assessed with a maximal cardiopulmonary exercise test and endurance cycling time during a constant work rate test at 65% of peak work rate, but endurance training was conducted at only 30%–40% of peak work rate, also interrupting training when peripheral oxygen saturation (SpO_2_) dropped below 90%. Other clinical trials seemed to employ outcome-training specificity (9,14–19,21–23).

## Exercise intensity: low moderate versus high

Exercise training intensity represents one of the key elements determining endurance training effects (33). Arguably, the main benefit of supplemental O_2_ is that it allows higher exercise intensities during the training program. To take advantage, exercise training with supplemental O_2_ must be performed at higher intensities compared with training in room air or even highest intensities, with subjects pushed to near their physiologic capabilities. In fact, high-intensity training is feasible in certain people with COPD who do not have contraindications, some of them are able to achieve also supramaximal work rates (i.e., 120%–140% of peak work rate) (34,35). In contrast, training programs employing “moderate/somewhat hard” intensities seem not influenced by supplemental O_2_ (9,20,27,28,30,32). Exploitation of supplemental O_2_’s benefits is achieved if the exercise intervention is based on an O_2_-specific training prescription leading to higher work rates and/or duration. Nine of 11 RCTs most frequently included in meta-analyses prescribed endurance training at high intensity (9,15–19,21–23), although its definition differed among studies; only Spielmanns and colleagues reached supramaximal work rate intensities, however excluding patients with EID (22).

## Exercise modality: continuous versus interval training

High-intensity interval training alternates short exercise bouts at near peak capacity with short bouts of rest or lower intensity. This modality enables (i) reaching high training work rates, thus exploiting additional supplemental O_2_ benefits, and (ii) maintaining a breathing reserve, (iii) thereby forestalling premature dyspnea and leg discomfort (36,37). Indeed, the interval-based training can avoid an overloading of the cardiorespiratory system, which, otherwise, could lead to an aggravation of air flow limitations and potentially dynamic hyperinflation (38–40). Therefore, high-intensity interval training might be preferred over continuous training, also because of a better tolerance. Three mentioned RCTs used this training methodology, whereas only Neunhäuserer et al. (18) found significant benefits of O_2_ supplementation. Spielmanns et al. and Wadell et al., either including normoxemic subjects (22) or stopping exercise if SpO_2_ dropped below 90% (23), did not demonstrate advantage for supplemental O_2_. However, considering the high prevalence of stable EID in this population, applying SpO_2_ drops below 90% as training discontinuation criterion, particularly when reached only during short bouts of high-intensity intervals, may hinder an effective increase of exercise intensity. Indeed, the most recent RCT of Alison et al. (14) interrupted training only for SpO_2_ <80%.

## Prescribing and progressing exercise intensity

When endurance training is programmed, HR, cycling work rate, and walking speed inclination as well as perceived effort and dyspnea are parameters used to prescribe exercise intensity. Most RCTs prescribed training interventions as a % of peak work rate derived from a cardiopulmonary exercise test (9,16,18,20–22), speed measured with the 6-min walking test (14), or %V̇O_2peak_ estimated with the Incremental/Endurance Shuttle Walk test (15,17,19). In most studies, exercise intensity was increased during the training program, in some according to objective criteria, in others “as tolerated” by assessment of the subject and/or the supervising therapist. Selection of methods to analyze exercise capacity, provide target exercise intensity, and ensure progressive training adaptations varied between studies. This criterion was considered in the TESTEX scale for quality assessment of exercise training studies (41). Overall, 6 of 11 RCTs explored in this review adjusted work rate over the course of the program (9,14,18,20,22,23), whereas 2 studies did not adjust (16,19), and 3 did not report this information (15,17,21).

## Exercise work rate adjustment: between-group comparisons

Adequate intensity and progression are main determinants of training intervention effectiveness. To take advantage of higher-intensity training not otherwise achievable without O_2_, exercise intensity must be adjusted between groups throughout the intervention period, enabling an increase of intensity in the supplemental O_2_ training group compared with that breathing room air (4,9,12). Interestingly, only Emtner et al. (9) and Alison et al. (14) reported these data. However, only the former showed higher work rates for the O_2_ group compared with the air group, whereas Alison et al. reported lower rating of perceived exertion and dyspnea during treadmill walking at moderate intensity, thus presumably under-dosing training intensity for the O_2_ group. In contrast, Neunhäuserer et al., although not providing work rate progression for each group, instituted gas-specific training prescriptions that might be expected to yield similar relative, but higher absolute work rates in the supplemental O_2_ group. Nine studies did not report training work rates (15–23). Indeed, “exercise volume and energy expenditure” is another criterion considered in the TESTEX scale for quality assessment of exercise training studies (41). When programming training at similar work rates, it should not be surprising to find no advantage of supplemental O_2_ compared with air-breathing exercise (24,42).

## Possible advantages of training in room air in nonhypoxemic COPD

Because evidence is still limited, current guidelines do not focus on exercise training interventions with supplemental O_2_ for nonhypoxemic COPD patients (3,8,10,11). It has been shown that cardiovascular and metabolic training adaptations can be reached without supplemental O_2_ (2,9,14,15,19,20,22,23,35,43). Furthermore, exercise training showed beneficial effects on systemic inflammation and endothelial dysfunction in COPD, which were not affected by use of supplemental O_2_ during the training program; some data seem to favor exercise training in room air to improve peripheral endothelial function (44). Thus, it might be useful to investigate how to best weigh advantages of normoxic and hyperoxic exercise training in nonhypoxemic COPD based on patients’ clinical conditions and predefined objectives.

## DISCUSSION

### Putting it all together and future study perspectives

**FIGURE 1:**
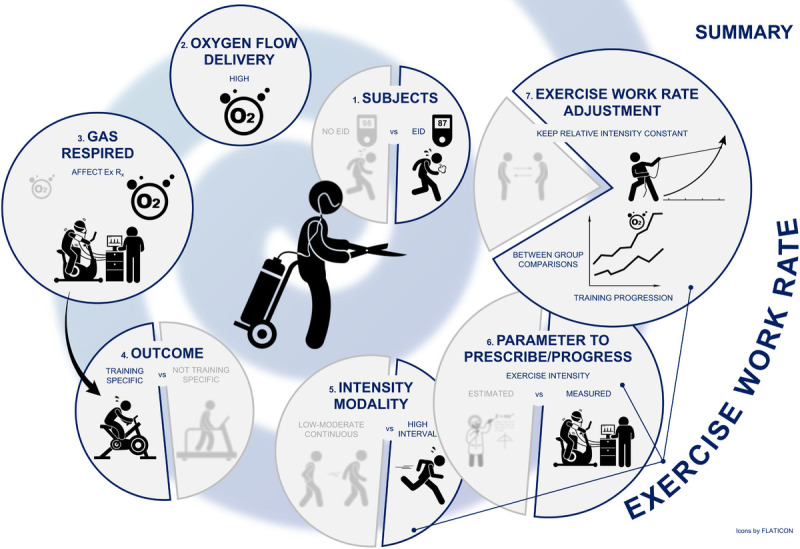
Summary of recommended cornerstone characteristics to consider for exercise prescription with oxygen supplementation in patients with COPD. Ventilatory limitations in COPD lead to dyspnea during physical exercise. Therefore, many patients are physically inactive leading to deconditioning and further worsening of functional capacity and thus quality of life. This deteriorating spiral can be interrupted by exercise training, but it is necessary to consider strategies to overcome these ventilatory limitations during exercise in order to ensure effective training stimuli. Supplemental oxygen is one of the available options for certain patients. When prescribing and applying physical exercise with supplemental oxygen: 1. Choose the right subject (EID and/or “responders”); 2. Apply an adequate oxygen flow; 3. Test patients with supplemental oxygen to prescribe appropriately; 4. Train specifically based on goals and outcome measures; 5. Consider oxygen supplementation particularly in case of high intensity; 6. Prescribe based on measured exercise parameters; 7. Adjust work rate progressively. EID, exercise induced desaturation; E_x_ R_x_, exercise prescription.

Exercise training is a ubiquitously available, safe and effective therapeutic intervention with considerable clinical benefit in COPD. Although scientific evidence seems limited, previously conducted exercise training interventions employing supplemental O_2_ present with a risk of interpretation bias when conducting pooled (meta-)analyses. It can be argued that, when applied with consideration to physiologic principles, training effects might be more pronounced when exercise is performed with supplemental O_2_ (Fig. 1).

Higher oxygen delivery in concentration and flow rate may safely facilitate a positive impact on the training intervention. Outcome-specific training with progressive adaptations of work rates leading to higher training intensities with supplemental O_2_ is a key design issue. Pretraining testing with room air as opposed to O_2_ will influence test results and thus affect training prescription. Supplemental O_2_ seems particularly relevant for high-intensity training programs where patients experience ventilatory limitations and peripheral muscle fatigability. It seems, however, worth considering whether high-intensity continuous training is routinely feasible in this population (9,15,16,19,21,33). Moreover, the impact of supplemental O_2_ has not yet been adequately investigated regarding different types of exercise programs and for specific COPD patients characteristics (25). It could be speculated that patients who desaturate during exercise and/or with limb muscle dysfunction may particularly benefit from supplemental O_2_, which might also be considered to facilitate gain in peripheral muscle mass and quality (25). It seems also useful for very deconditioned and/or dyspneic patients in early stages of rehabilitation to promote more rapid increase in exercise capacity (18,25). In contrast, patients with endothelial dysfunction might not be the best candidates for O_2_ supplementation (44).

Current “pooled analyses” provide fewer specific insights and may somehow not seem in line with the potential impact of supplemental O_2_ described in this scientific analysis of current literature, also because of some study limitations and biases in underlying original trails. Indeed, some common shortcomings are related to low quality in study reporting, which should be considered in the final data interpretations (especially criteria 11 and 12 of the TESTEX scale).

Because there is currently limited evidence supporting these hypotheses, well-designed clinical exercise training trails are needed to avoid misleading conclusions for this important training adjunct for COPD and other pulmonary disorders.

### Limitations and perspectives

Given the nature of this brief narrative review, the applied research methodology was not as rigorous as in a systematic review or meta-analysis leading to pooled outcomes, but it allowed more specific, still standardized, analyses of the underlying original studies, calling for future well-designed clinical trials. Second, not all evaluated studies reported all parameters considered in this manuscript as cornerstone characteristics; therefore, this methodological and reporting bias should also be carefully addressed in upcoming RCTs on this topic.

## CONCLUSIONS

In this review, we provide an exercise physiology focused analysis of current evidence. Applications of supplemental O_2_ during training interventions in COPD have been explored, which need corroboration by more scientific evidence.

The main conclusion is that hypothesis regarding the impact of supplemental O_2_ during exercise training in COPD should be modified: future studies should not simply focus on whether training with supplemental O_2_ is more effective than in room air, but rather to understand when, how and in which patients it could be used to reach specific training goals. How can physiologic advantages from hyperoxic exercise training be exploited for these patients, with outcome-specific training targets and progressive work rate adaptations? Supplemental O_2_, considering a higher concentration or flow rate, might be specifically prescribed for certain responding patients and in specific clinical conditions, when high-intensity training is desired and not contraindicated, with the aim of improving pulmonary rehabilitation outcomes on exercise capacity and endurance.
